# Renal transplantation in older recipients – results of the DZIF transplant cohort

**DOI:** 10.1186/s12877-026-07901-0

**Published:** 2026-07-06

**Authors:** Claudia Sommerer, Iris Schröter, Daniela Schindler, Anja Schork, Lutz Renders, Stephan Kemmner, Jonas Michael Willerding, Burkhard Tönshoff, Martin Zeier, Thomas Giese

**Affiliations:** 1 Department of Nephrology, University Hospital Heidelberg, Im Neuenheimer Feld 162, Heidelberg, 69120 Germany; 2 Department of Nephrology, Klinikum Rechts der Isar of the Technical University Munich, Munich, Germany; 3 Department of Diabetology, Endocrinology, University Hospital Tuebingen, Tuebingen, Nephrology Germany; 4 Transplant Center, LMU University Hospital, LMU Munich, Munich, Germany; 5 Department of Nephrology, Hannover Medical School, Hannover, Germany; 6 Department of Pediatrics I, University Children’s Hospital Heidelberg, Heidelberg, Germany; 7 Department of Immunology, University Hospital Heidelberg, Heidelberg, Germany; 8 German Centre for Infection Research (DZIF), Heidelberg Site, Heidelberg, Germany

**Keywords:** Renal transplantation, Age, Mortality, Infection

## Abstract

**Background:**

Kidney transplantation in older adults is expanding, but detailed data on infection burden, antibiotic resistance, and sex-specific outcomes remain limited. The aim of the present study was to characterize infection dynamics and determinants of adverse events in recipients aged ≥ 65 years.

**Methods:**

In this multicenter cohort study, 355 kidney transplant recipients aged 65–80 years (67.6% male) were followed for a median of 3.8 years. Cumulative incidences of infection, graft loss, and death were determined. Independent risk factors were identified using Cox regression analyses.

**Results:**

Delayed graft function occurred in 23.9%, S-creatinine was 1.72 IQR (1.40–2.45) and eGFR 38.7 (26.2–51.3) one year after transplantation. All-cause mortality was 3.2% at year 1 and 11.8% at year 5 after transplantation. Infectious complications were frequent, with a cumulative incidence of first infection reaching 67.6% at 1 year and 81.9% at 5 years. Bacterial infections predominated, whereas viral infections persisted throughout follow-up and fungal infections occurred mainly in the early post-transplant period. During follow-up, death with a functioning graft (DWFG) was the predominant graft-related endpoint, accounting for 35 of 48 all-cause graft loss events, with infections representing the leading attributed cause (51.4%). In multivariable analyses, prolonged initial hospital stay was consistently associated with infectious outcomes and subsequent DWFG, while delayed graft function was independently associated with increased hazard of DWFG (HR 2.54, 95% CI 1.29–5.00). In time-dependent analyses, fungal infections were independently associated with a higher subsequent hazard of DWFG (HR 2.67, 95% CI 1.23–5.77). Mortality, graft-related outcomes, and infection rates were broadly similar by sex and between recipients aged 65–69 and ≥ 70 years.

**Conclusions:**

Kidney transplantation in carefully selected older recipients, including those aged ≥ 70 years, was associated with sustained patient and graft survival. Infectious complications occurred frequently and constituted the leading attributed cause of DWFG; this underscores the importance of understanding why some recipients recover from infectious stress, while others experience subsequent clinical deterioration.

**Supplementary Information:**

The online version contains supplementary material available at 10.1186/s12877-026-07901-0.

## Introduction

Increasing life expectancy in the Western world leads to a growing number of older patients with end-stage renal disease [1, 2]. Internationally, the need for renal replacement therapy is increasing most rapidly in this age group [3, 4]. In Germany, another contributing factor is the long waiting time of eight to ten years on average for a kidney transplant, which means that older people are often already quite old by the time they are offered a transplant [5]. As a result, recipients aged 65 years and older represent a rapidly expanding population in contemporary transplant practice.

In clinical practice, transplant teams face complex decisions regarding eligibility, donor selection, and post-transplant management in patients aged ≥ 65 years. Although transplantation can offer significant advantages over dialysis in terms of survival and quality of life, older recipients represent a particularly vulnerable patient group in whom outcomes are strongly influenced by comorbidities, functional reserve, cognitive function and susceptibility to complications [6, 7]. Wolfe et al. showed that patients aged 60 to 74 years had a 61% reduction in mortality and a four-year increase in life expectancy [8]. Rao et al. demonstrated that transplant recipients over 70 years of age had a 41% lower overall mortality risk than dialysis-dependent patients on the waiting list [9]. Even older patients with comorbidities such as diabetes and hypertension as the cause of end-stage renal disease also experienced a large benefit. However, the transplantation was associated with an initial increase in mortality during the first three months [9]. Beyond chronological age, older transplant candidates frequently exhibit cardiovascular disease, hypertension, diabetes, a history of cancer, frailty, and polypharmacy. Age-related immune dysfunction further alters host defense mechanisms and may affect tolerability of immunosuppressive therapy. At the same time, older recipients often receive organs from older donors or donors with extended criteria, which may complicate early graft function and perioperative management. Furthermore, clinicians must operate within a narrow therapeutic window, balancing the prevention of rejection against the risk of infection in patients with limited physiological reserve. Infections remain one of the most common causes for hospitalization after kidney transplantation and contribute significantly to morbidity and mortality [10–16]. Older recipients may be particularly susceptible due to age-related immune dysfunction including immunosenescence [17–19]. On the other hand, immunosenescence could be an important factor in transplant tolerance [20].

Despite the growing number of older transplant recipients, real-world data describing clinical outcomes and infection patterns specifically in this age group remain limited.

As transplantation programs such as the Eurotransplant European Senior Program increasingly care for older recipients and utilize older donor organs, clinicians require reliable multicenter data to identify relevant risks and develop individualized strategies for immunosuppression, infection prevention, and post-transplant care in this patient cohort [21]. The aim of this study was to evaluate patient survival, graft outcomes, and infectious complications in kidney transplant recipients aged ≥ 65 years within the framework of a current multicenter transplant cohort study.

## Methods

The DZIF transplant cohort is a prospective, multicenter cohort study conducted by the German Center for Infection Research [13, 22]. It includes allograft recipients from five of the largest German transplant centers (University Hospital Hannover, University Hospital and Renal Center Heidelberg, TU Munich, LMU Munich, and University Hospital Tuebingen). All organ recipients at the participating transplant centers were informed about the study and invited to participate.

In the present study, we analyzed data from adult kidney allograft recipients aged 65 and older who provided written informed consent to participate and received a transplant between April 2014 and November 2025. The ethics committees of all participating centers granted their approval (Hannover Medical School Nr 6534, Medical Faculty of the University of Heidelberg Nr S-585/2013, Medical Faculty of the TU Munich Nr 5926/13, LMU Munich Nr 380–15, University Hospital Tuebingen Nr 327/2014BO1), and all participants provided written informed consent.

Clinical events were systematically recorded and evaluated. Study visits took place immediately before transplantation and at 3, 6, 9, and 12 months post-transplantation. Annual follow-up visits were also conducted, or as clinically necessary in cases of infectious events. Clinical, laboratory, and demographic data were collected from medical records.

### Immunosuppression and prophylaxis strategy

Standard immunosuppression consisted of prednisolone, mycophenolic-acid, tacrolimus, and an induction therapy with basiliximab [23]. Participating transplant centers followed a standard concept with a suggested initial Tac C0 level of 6–10 µg/l in the first 3 months, followed by a Tac C0 level of 5–8 µg/l until month 6. Thereafter, Tac was stepwise reduced to a Tac C0 level of 4–6 µg/l in the long-term. Mycophenolic acid was administered in a standard dosage of mycophenolate mofetil 1000 mg or 720 mg mycophenolic acid twice a day. In case of certain complications as leucopenia, diarrhea, recurrent infections the dosage was reduced.

Prophylaxis and monitoring were performed according to the KDIGO 2009 guidelines. For Pneumocystis jirovecii prophylaxis, trimethoprim-sulfamethoxazole (800 mg/160 mg) was routinely administered three times a week for the first 6 months after transplantation. Standard antiviral prophylaxis with valganciclovir was administered to all Cytomegalovirus (CMV) immunoglobulin G (IgG)–positive recipients and recipients of CMV IgG–positive donor organs for at least 3 months. In high-risk (D +/R–) cases, 6 months of antiviral prophylaxis was recommended. The dosage was adjusted according to renal function. Candida prophylaxis with oral nystatin was provided within the first one to 3 months if more than 20 mg of methylprednisolone was administered daily.

Routine postoperative management included temporary urinary drainage and ureteral stenting in accordance with the centers´ local protocols.

### Clinical definitions

All infections requiring hospitalization were included. Diagnoses were made by the treating physician. Data collected included clinical presentation, laboratory and microbiological/virological findings, diagnostic procedures, treatment, disease course, and infection-related outcomes. Detailed infection definitions are provided in the Supplementary Table S1.

Delayed graft function (DGF) was defined as the requirement for dialysis within the first 7 days after transplantation, excluding dialysis performed solely for hyperkalemia, volume overload, or other non–graft-related indications.

Intermediate care unit (IMC) stay was defined as the duration of routine postoperative treatment in the surgical intermediate care unit following kidney transplantation and was recorded in days. At the participating centers, patients were routinely transferred to the surgical IMC immediately after transplantation as part of standard postoperative care.

### Statistical methods

Continuous variables are presented as median with interquartile range (IQR), and categorical variables as counts and percentages. Group differences in baseline characteristics were asseses using the Mann–Whitney U test for continuous variables and the χ^2^ test or Fisher’s exact test, as appropriate, for categorical variables.

Time-to-event outcomes included all-cause mortality, all-cause graft loss, death-censored graft loss (DCGL), death with a functioning graft (DWFG), and first infection. Additional pathogen-specific analyses were performed separately for first bacterial, first viral, first fungal, and first resistant infection, with each pathogen category analyzed independently.

Overall patient survival was estimated using the Kaplan–Meier method and compared by log-rank test. Cumulative incidence functions (CIF) were calculated descriptively for time-to-event outcomes, treating competing events according to the outcome under investigation.

Associations with time-to-event outcomes were assessed using cause-specific Cox proportional hazards regression models, with competing events treated as censoring events at the time of occurrence, as appropriate for the respective endpoint. Univariable analyses were first performed for clinically relevant baseline and peri-transplant covariates. Multivariable models were then constructed using a parsimonious variable selection strategy based on clinical relevance, univariable associations (*p* < 0.10), and the number of observed outcome events, in order to reduce the risk of overfitting. Hazard ratios (HRs) with 95% confidence intervals (CIs) are reported. To evaluate the temporal relationship between infection episodes and subsequent outcomes, bacterial, viral, and fungal infections were additionally modeled as time-dependent covariates in counting-process style Cox regression models. Patients contributed person-time as unexposed until the first occurrence of the respective infection type and as exposed thereafter, ensuring that only infections occurring before a given risk interval contributed to exposure status.

In a separate analysis, cumulative infectious burden was assessed by modeling the total number of documented bacterial, viral, and fungal infection episodes as a time-dependent cumulative count variable, which increased by one at the time of each infection episode during follow-up. Thus, the cumulative infection count was updated over time and entered as a dynamic exposure variable in Cox regression models, allowing assessment of whether increasing infectious burden during follow-up was associated with subsequent outcome risk.

Renal function was analyzed as an additional outcome using estimated glomerular filtration rate (eGFR) at approximately one year after transplantation, based on the laboratory value closest to day 365 post-transplant. Associations with eGFR were assessed using linear regression models, and clinically relevant renal dysfunction (eGFR < 45 mL/min/1.73m^2^) was additionally analyzed using logistic regression models.

Proportional hazards assumptions were assessed using Schoenfeld residuals. All analyses were considered exploratory. A two-sided *p* value < 0.05 was considered statistically significant.

All analyses were conducted using complete-case data. No imputation of missing values was performed. Therefore, denominators may vary across variables.

All analyses were performed using R (version 4.4.2).

## Results

A total of 355 kidney transplant recipients aged ≥ 65 years (maximum 80 years) were included in the analysis (Table 1). Median age was 67 years (IQR 66–70) with 95 patients being ≥ 70 years (26.7%, Table S1). 67.6% transplant recipients were male. Median follow-up was 3.8 years (IQR 1.8–6.5).

**Table 1:** Baseline characteristics in older renal allograft recipients by sex

Characteristic	Overall *N* = 355	Female recipients *N* = 115	Male recipients *N* = 240	*p*-value
Recipient characteristics				
Age at transplantation, years	67.0 (66.0–70.0)	67.0 (66.0–69.0)	67.0 (66.0–70.0)	0.567
Time of follow up, years	3.8 (1.8–6.5)	3.4 (1.4–6.0)	4.0 (1.9–6.7)	0.118
Body mass index, kg/m^2^	26.0 (23.7–28.7)	25.2 (22.3–29.3)	26.3 (24.2–28.4)	0.124
Primary kidney disease (top categories)				
Glomerulonephritis (combined)	81 (23.8%)	15 (13.5%)	66 (28.7%)	0.003
ADPKD	51 (15.0%)	22 (19.8%)	29 (12.6%)	0.104
Diabetes mellitus type 1	8 (2.3%)	4 (3.6%)	4 (1.7%)	0.282
Diabetes mellitus type 2	24 (7.0%)	3 (2.7%)	21 (9.1%)	0.040
FSGS	18 (5.3%)	5 (4.5%)	13 (5.6%)	0.799
Vasculitis/collagen vascular disease	13 (3.7%)	3 (2.7%)	8 (3.5%)	0.999
Transplant and procedure				0.571
retransplantation	38 (11.1%)	15 (13.5%)	23 (10.0%)	0.361
Multi-organ transplantation (pancreas–kidney)	8 (2.3%)	3 (2.6%)	5 (2.1%)	0.717
AB0 incompatibility	10 (3.0%)	1 (0.9%)	9 (3.9)	0.177
Recipient serology				
CMV IgG positive	198 (57%)	76 (67.3%)	123 (52.6%)	0.011
EBV IgG positive	288 (81.1%)	86 (74.8%)	202 (84.2%)	0.041
Donor characteristics				
Deceased donation	316 (89.5%)	105 (91.3%)	211 (88.7%)	0.578
Donor male gender	135 (41.4%)	40 (38.1%)	95 (43.0%)	0.470
Donor age class				0.374
< 60 years	30 (9.2%)	6 (5.8%)	24 (10.8%)	
60–74 years	180 (55.4%)	59 (7.3%)	121 (54.5%)	
≥ 75 years	115 (35.4%)	38 (36.9%)	77 (34.7%)	
Donor serology				
CMV IgG positive	199 (57.3%)	76 (67.3%)	123 (52.6%)	0.816
EBV IgG positive	288 (81.1%)	86 (74.8%)	202 (84.2%)	0.041
CMV donor/recipient constellation				0.031
D +/R +	125 (36.2%)	44 (39.3%)	81 (34.8%)	
D +/R −	81 (23.5%)	22 (19.6%)	59 (25.3%)	
D −/R +	74 (21.4%)	32 (28.6%)	42 (18.0%)	
D −/R −	65 (18.8%)	14 (12.5%)	51 (21.9%)	
Induction therapy				
intensified induction therapy	69 (19.4%)	25 (21.7%)	44 (18.3%)	0.471
Thymoglobulin	39 (11.0%)	14 (12.2%)	25 (10.4%)	0.999
Plasmapheresis	19 (5.4%)	11 (4.6%)	25 (10.4%)	0.999
Postoperative variables				
Delayed graft function (DGF)	85 (23.9%)	31 (27.0%)	54 (22.5%)	0.356
Inpatient days	19.0 (14.0–27.0)	17 (14–27)	19 (14–28)	0.342
Cold ischemia time, min	545.0 (394.0–750.0)	593 (443–754)	535 (387–739)	0.283
IMC days	5.0 (4.0–6.0)	5.0 (4.0–7.0)	5.0 (4.0–6.0)	0.616

Continuous variables are presented as median (interquartile range, Q1–Q3) and categorical variables as number (percentage). Percentages were calculated within each column using non-missing data. Analyses were based on complete cases; therefore, denominators may vary across variables

Comparisons were performed on complete cases using the Mann–Whitney U test for continuous variables and Fisher’s exact test for categorical variables

CMV donor/recipient constellations were defined as D +/R +, D +/R −, D −/R +, and D −/R −

*Abbreviations: ADPKD* autosomal dominant polycystic kidney disease, *BMI* body mass index, *CIT* cold ischemia time, *CMV* cytomegalovirus, *DGF* delayed graft function, *EBV* Epstein–Barr virus, *FSGS* focal segmental glomerulosclerosis, *IMC* intermediate care unit, *D* donor, *R* recipient

Most patients underwent their first kidney transplantation (88.9%). The most frequent causes of end-stage kidney disease were IgA nephropathy (18.8%), autosomal dominant polycystic kidney disease (15.0%), and diabetic nephropathy (9.2%). Living kidney donation was performed in 10.5%. Early post-transplant parameters showed a median hospital stay of 19 days (IQR = 14–27), and median intermediate care unit stay of 5 days [4–6]. Delayed graft function occurred in 23.9% of recipients.

Similarly, baseline characteristics were well balanced between female (*n* = 115) and male recipients (*n* = 240), Table 1. Female recipients were more frequently CMV IgG seropositive (67.3% vs. 52.6%, *p* = 0.011), whereas male recipients showed higher EBV IgG seropositivity (84.2% vs. 74.8%, *p* = 0.041). Primary kidney disease differed by sex, with a higher prevalence of autosomal dominant polycystic kidney disease among females (19.8% vs.12.1%, *p* = 0.102), and higher rates of IgA nephropathy (8.1% vs.23.9%, *p* < 0.001) and type 2 diabetes (9.1% vs. 2.7%, *p* = 0.040) among males.

### Overall clinical outcomes

**Fig. 1:**
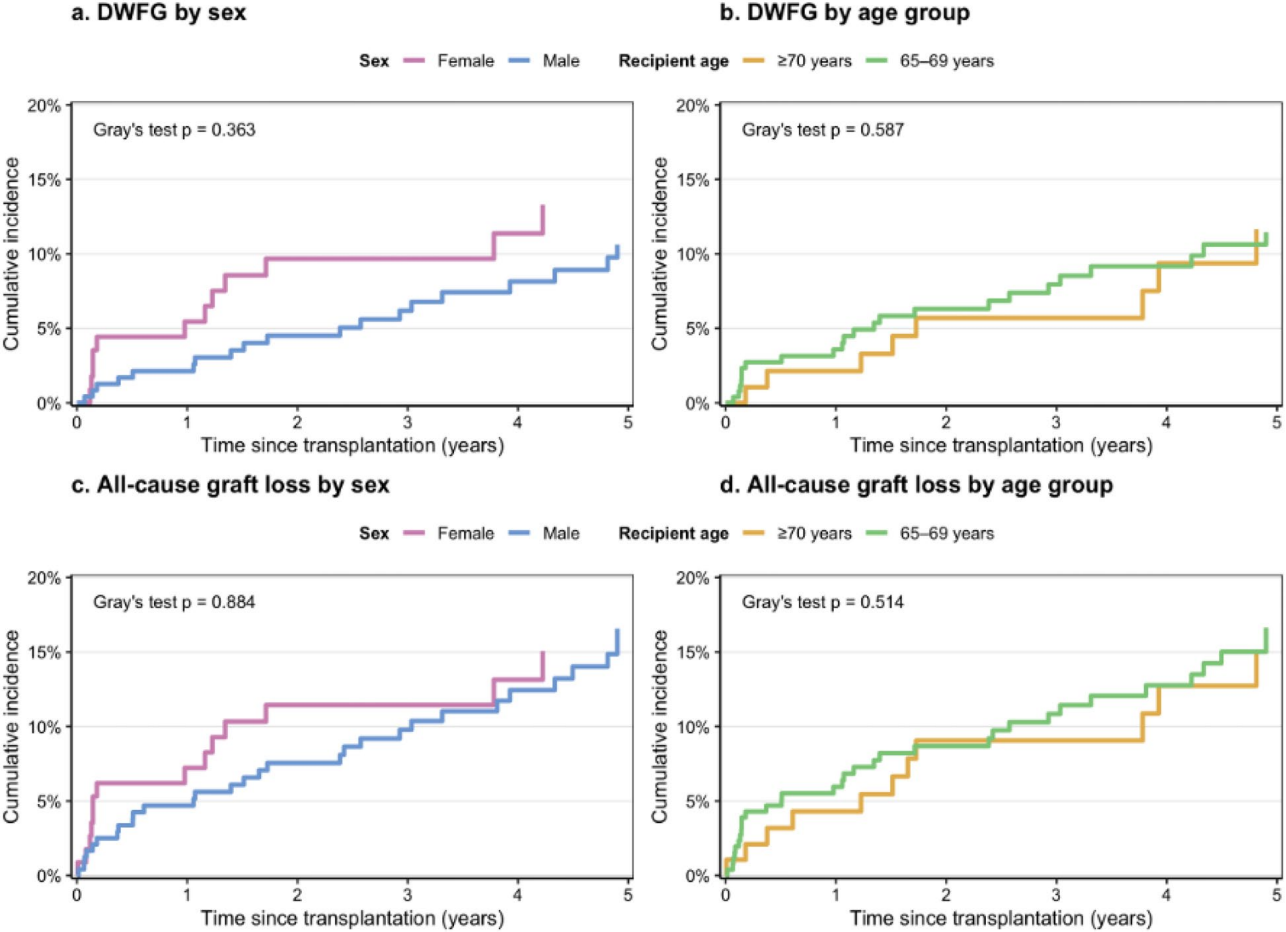
Cumulative incidence of graft-related outcomes according to recipient sex and age group. Cumulative incidence functions for death with a functioning graft (DWFG) stratified by recipient sex (**a**) and recipient age group (**b**), and all-cause graft loss stratified by recipient sex (**c**) and recipient age group (**d**). Group comparisons were performed using Gray’s test. DWFG, death with a functioning graft

In the overall cohort of recipients aged ≥ 65 years, delayed graft function occurred in 23.9%, S-creatinine was 1.72 IQR (1.40–2.45) and eGFR 38.7 (26.2–51.3) one year after transplantation. All-cause mortality was 3.2% at year 1 and 11.8% at year 5 after transplantation. The cumulative risk of all-cause graft loss increased from 5.7% at 1 year to 10.7% at 3 years and 16.9% at 5 years (Table 2). Of the 48 all-cause graft loss events, 35 represented DWFG, whereas 13 were DCGL. At 5 years, cumulative incidence was 11,2% for DWFG and 4,4% for DCGL (Table 2). Corresponding cumulative incidence curves are shown in Fig. 1. Among DWFG cases, infections were the leading cause (51.4%), followed by cardiovascular causes (11.4%) and malignancy-related deaths (8.6%). Among DCGL cases, rejection-related graft loss was the most common cause (30.8%), followed by acute-on-chronic or multifactorial graft failure (30.8% combined). A detailed breakdown of underlying causes of DWFG and DCGL is provided in Tables 3 and 4, respectively. Corresponding cumulative incidence function curves are shown in Fig. 1.

**Table 2:** Cumulative incidence of graft-related outcomes at 1, 3, and 5 years

Outcome	Time	Cumulative incidence (%)	95% CI
All cause-mortality	1 year	3.2	1.3–5.1
	3 years	7.5	4.5–10.4
	5 years	11.8	7.6–15.8
All-cause graft loss	1 year	5.5	3.1–7.9
	3 years	10.4	6.9–13.7
	5 years	16.2	11.5–20.7
Death with functioning graft (DWFG)	1 year	3.0	1.2–4.8
	3 years	7.1	4.3–10.0
	5 years	11.2	7.3–15.1
Death-censored graft loss (DCGL)	1 year	2.2	0.6–3.7
	3 years	2.8	1.1–4.6
	5 years	4.4	1.9–6.9

Values represent cumulative incidence estimates at 1, 3, and 5 years. All-cause mortality and graft loss was estimated using the Kaplan–Meier method. Death with a functioning graft (DWFG) and death-censored graft loss (DCGL) were estimated using competing-risk cumulative incidence functions, treating each event type as a competing event for the other

*CI* confidence interval, *DWFG* death with functioning graft, *DCGL* death-censored graft loss

**Table 3:** Underlying causes of death with functioning graft (DWFG)

Cause	n	%
Infection-related death	**18**	**51.4%**
Cardiovascular death	4	11.4%
Malignancy-related death	3	8.6%
Neurological/cerebrovascular death	1	2.9%
Therapy-related complication	1	2.9%
Sudden unexplained/non-malignant unspecified	4	11.4%
Unknown/unavailable	4	11.4%
Total	**35**	**100%**

**Table 4:** Underlying causes of death-censored graft loss (DCGL)

Cause	n	%
Rejection-related graft loss	**4**	**30.8**
Acute-on-chronic graft failure	2	15.4
Multifactorial graft failure	2	15.4
Recurrence of underlying disease	1	7.7
Primary non-function	1	7.7
Surgical complication/transplant nephrectomy	1	7.7
Cause unavailable	3	23.1
Total	**13**	**100**

During follow-up, 11.5% of recipients (41/355) developed a de novo malignancy. Skin cancers accounted for the majority of cases (80.5% [33/41]), followed by solid organ tumors (14.6% [6/41]), with one case of EBV-positive post-transplant lymphoproliferative disorder (PTLD). Malignancies occurred more frequently in male than female recipients (13.3% [32/240] vs 7.8%) [9/115].

### Infection burden and temporal distribution

**Fig. 2:**
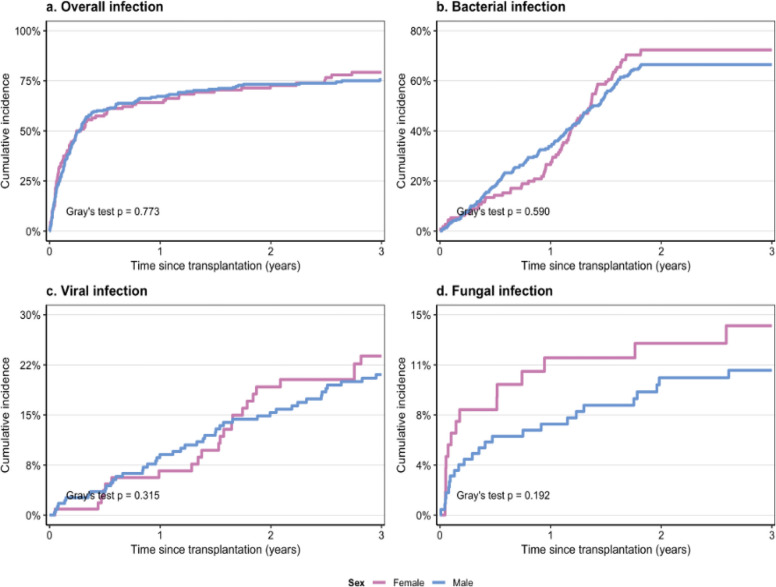
Cumulative incidence of first infectious complications after kidney transplantation according to recipient sex. Cumulative incidence functions for first (**a**) overall, **b** bacterial, **c** viral, and **d** fungal infections within 3 years after transplantation in female and male recipients. Each pathogen category was analyzed separately, irrespective of whether another infection type had occurred previously.Death and graft loss were considered competing events. Group comparisons were performed using Gray’s test

**Fig. 3:**
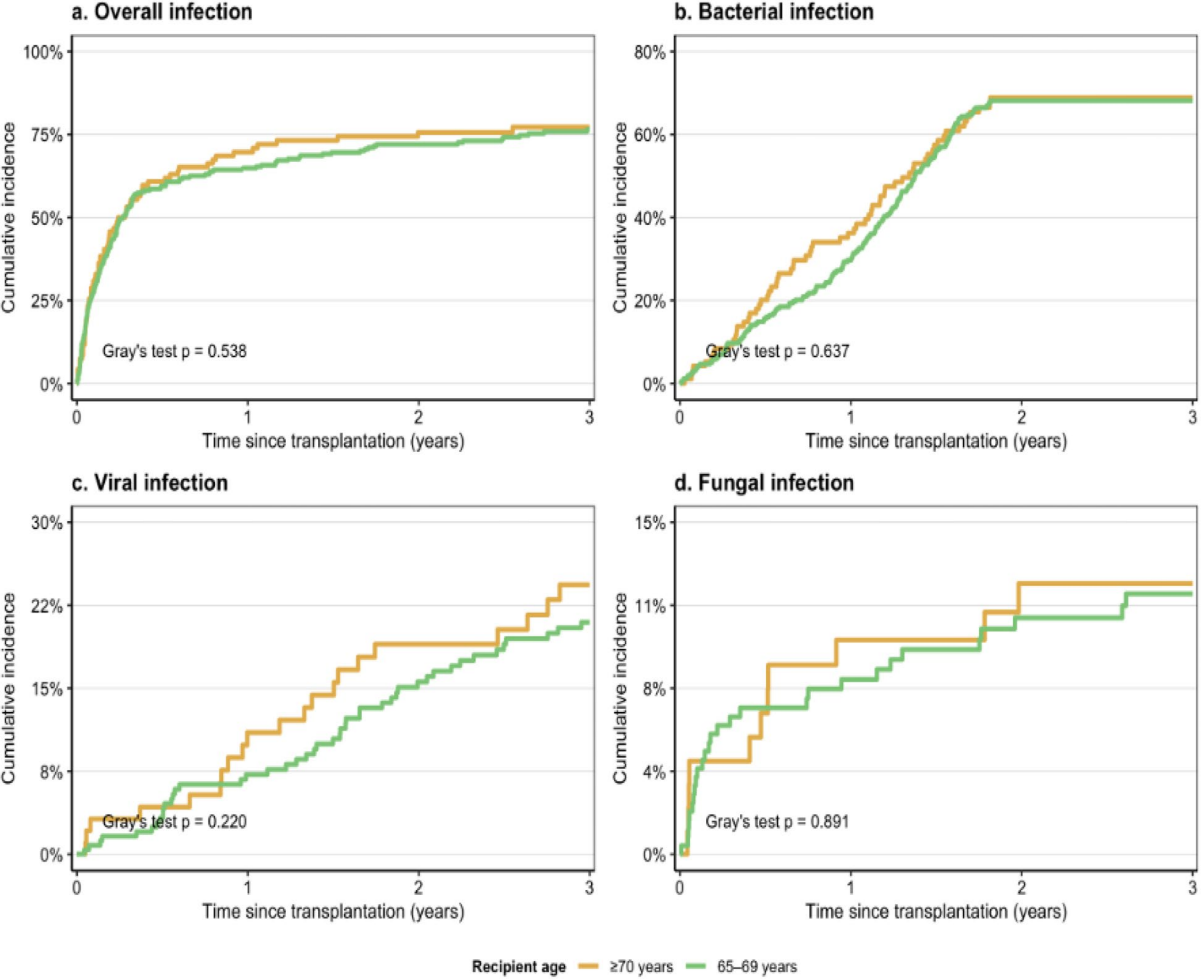
Cumulative incidence of first infectious complications after kidney transplantation according to recipient age. Cumulative incidence functions for first (**a**) overall, **b** bacterial, **c** viral, and **d** fungal infections within 3 years after transplantation in recipients aged 65–69 years and ≥ 70 years. Death and graft loss were considered competing events. Group comparisons were performed using Gray’s test

The cumulative incidence of first infection was high early after transplantation, reaching 50.5% (95% CI 44.8–55.5) at 3 months and 60.9% (95% CI 55.3–65.9) at 6 months, and further increasing to 67.6% (95% CI 62.0–72.3) at 1 year, 78.7% (95% CI 73.4–82.9) at 3 years, and 81.9% (95% CI 76.6–86.0) at 5 years, Figs. 2 and 3. Beyond first-event analyses, the overall infectious burden during follow-up was substantial. Across 1,468 patient-years, 938 infection episodes were documented in 257 recipients, corresponding to an overall rate of 63.9 episodes per 100 patient-years, with bacterial infections accounting for the largest proportion. On a patient level, recipients experienced a median of 3 infection episodes (IQR 2–5) during follow-up. Detailed pathogen-specific infection frequencies and rates are provided in Supplementary Table S2.

**Fig. 4:**
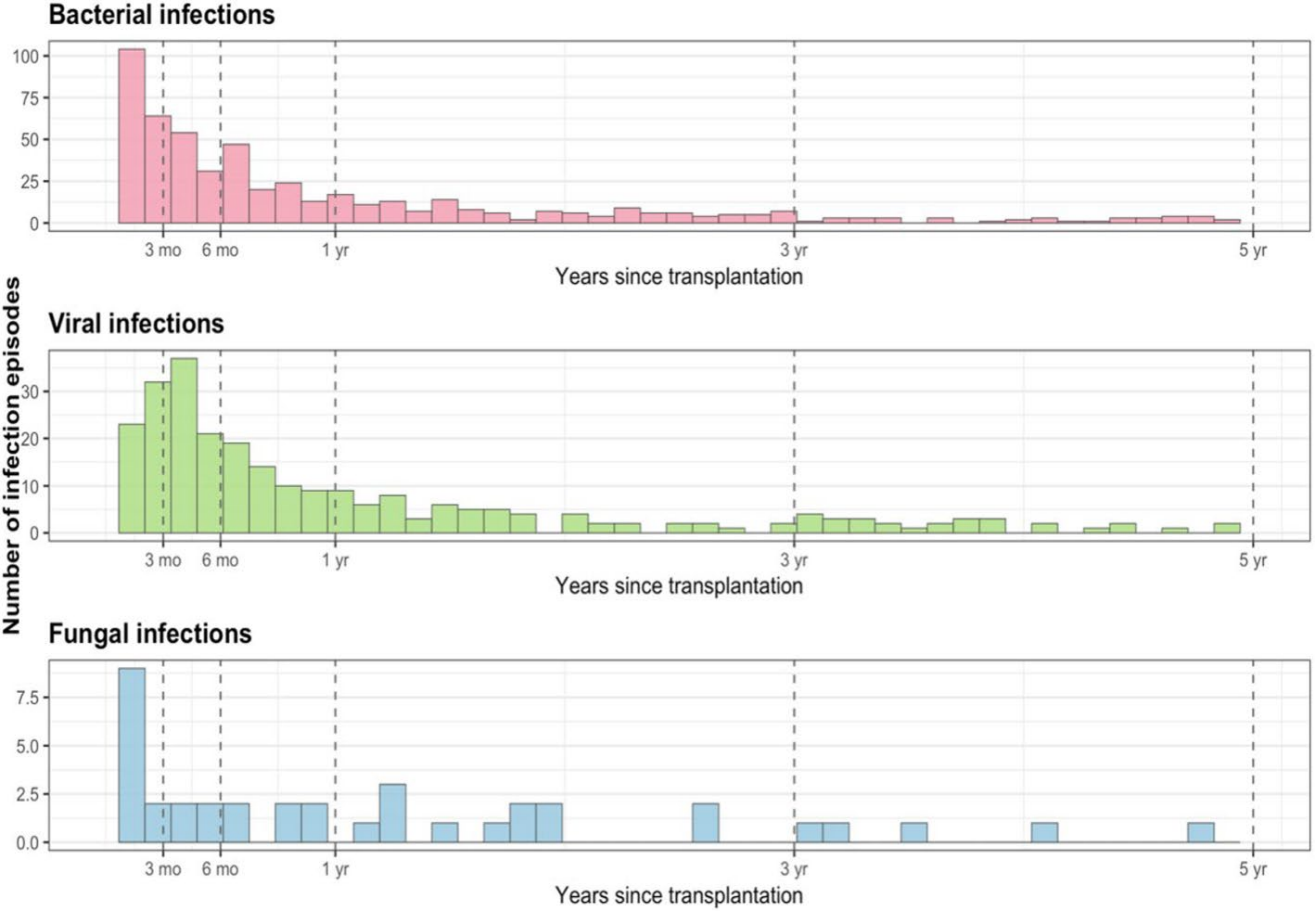
Timing of bacterial, viral, and fungal infections after transplantation. Histograms display the distribution of infection episodes over time following transplantation, stratified by pathogen class (bacterial, viral, fungal). The x-axis represents years since transplantation. Vertical dashed lines indicate clinically relevant time points (3 months, 6 months, 1 year, 3 years, and 5 years)

**Fig. 5:**
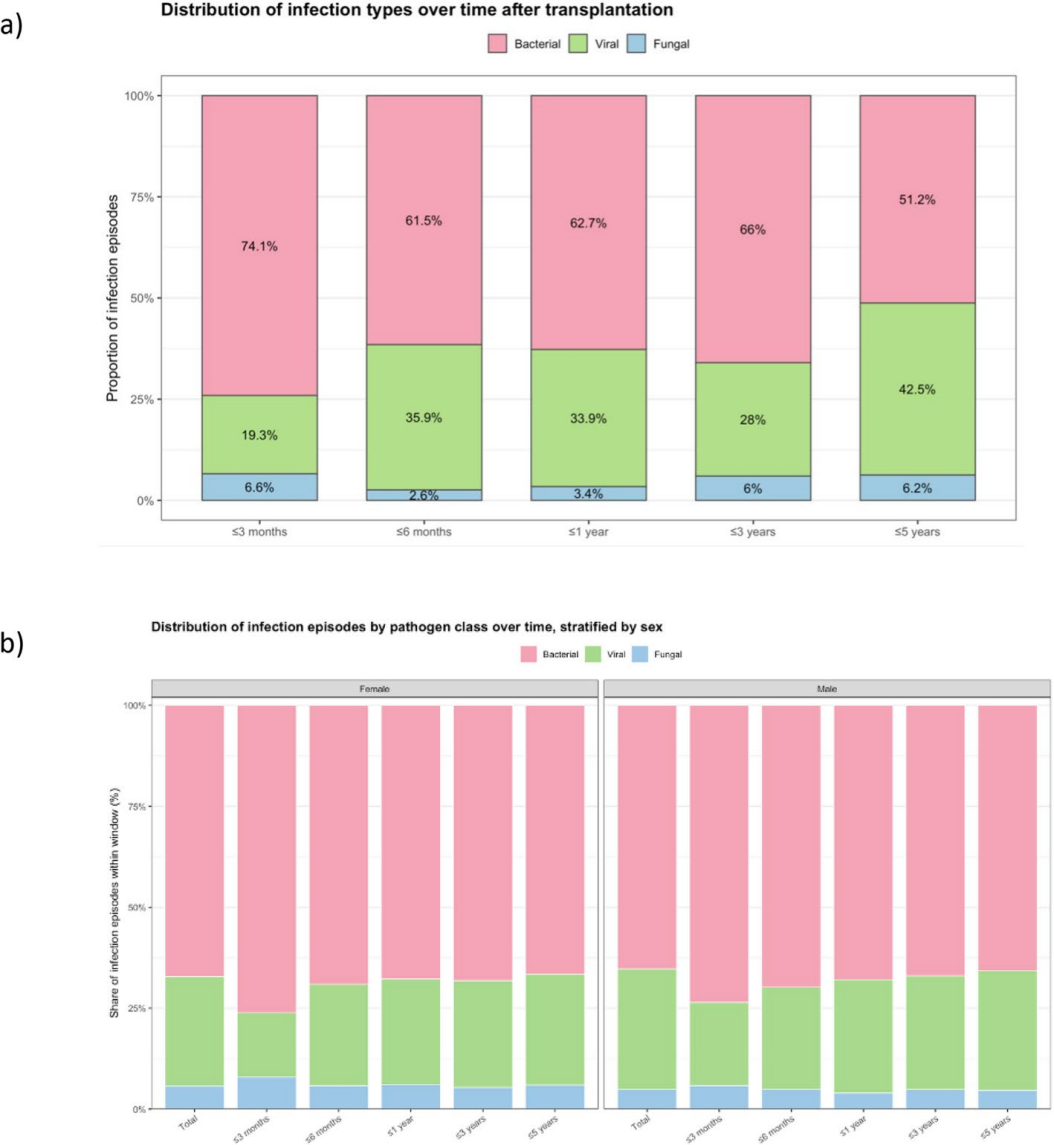
Distribution of infection types over time after transplantation in **a**) all allograft recipients, and **b**) in female and male allograft recipients. Stacked bar charts illustrate the proportional distribution of bacterial, viral, and fungal infection episodes at predefined time intervals after transplantation (≤ 3 months, ≤ 6 months, ≤ 1 year, ≤ 3 years, ≤ 5 years). Percentages represent the relative contribution of each infection type within each time window

Infection episodes clustered predominantly within the early post-transplant period, particularly for bacterial infections, which peaked within the first 3 months after transplantation, Fig. 4. Viral infections showed a broader temporal distribution, with substantial numbers occurring beyond 6 months and continuing throughout long-term follow-up, Fig. 5. Fungal infections were comparatively rare and occurred mainly early after transplantation, although sporadic late events were observed up to 5 years. Across all infection episodes, bacterial infections accounted for 66.0%, viral infections for 28.9%, and fungal infections for 5.2%. In the first 3 months post-transplantation, bacterial infections predominated (74.5%), while viral infections accounted for 18.9% and fungal infections for 6.6%. Over time, the relative proportion of viral infections increased (27.6% by 3 years; 28.9% by 5 years), whereas bacterial infections remained the majority (~ 66%). Median time to first infection was 3.0 months. Fungal infections consistently represented a small proportion of episodes throughout follow-up (2.6–6.6%).

### Bacterial infections

**Fig. 6:**
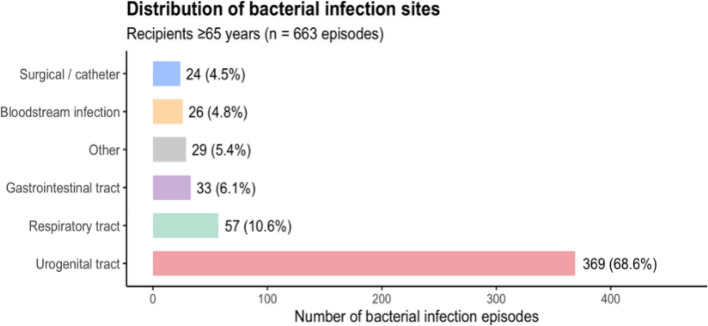
Distribution of bacterial infection sites in kidney transplant recipients aged ≥ 65 year. Among 663 bacterial infection episodes, urogenital infections predominated, followed by respiratory tract, bloodstream, gastrointestinal, and surgical/catheter-related infections. Percentages refer to proportions of all bacterial episodes

Among 663 bacterial infection episodes, E.coli (21.0%) and Enterococcus spp. (19.9%) were the most frequently identified pathogens, followed by Klebsiella spp. (11.5%) and Pseudomonas aeruginosa (11.3%). Urinary tract infections were by far the most common site, accounting for 68.5% of all episodes. Respiratory tract infections comprised 10.6%, followed by bloodstream infections (4.8%) and gastrointestinal infections (6.1%). Surgical site and catheter-related infections accounted for 4.5%, whereas all other infection sites individually represented less than 1% of episodes, Fig. 6 and S1. Bacterial infection sites over time were comparable between age groups (Fig. 2S).

Overall, multidrug-resistant organisms were identified in 89 episodes (13.3%). MRGN (3/4MRGN) was identified in 48 episodes (7.2%), including 36 (5.4%) classified as 3MRGN and 12 (1.8%) as 4MRGN. VRE was detected in 33 episodes (5.0%), MRSA in 4 (0.6%), and MDR in 3 (0.5%). ESBL-producing organisms were rare (1 episode, 0.2%).

### Viral infections

A total of 287 viral infection episodes were documented in the study cohort. Cytomegalovirus (CMV) was the most frequently identified pathogen, accounting for 102 episodes (35.5%). BK virus was detected in 61 episodes (21.3%). Respiratory viruses accounted for 90 episodes (31.4%) of all documented viral infections. Other members of the Herpesviridae family were less frequent: herpes simplex virus type 1 (HSV-1) was identified in 17 episodes (5.9%), varicella zoster virus (VZV) in 12 (4.2%), herpes simplex virus type 2 (HSV-2) in 5 (1.7%), and Epstein–Barr virus (EBV) in 1 episode (0.3%). Norovirus was detected in 3 episodes (1.0%), and hepatitis E virus in 5 cases (1.7%). Hepatitis A–D viruses, HIV, and several other screened viruses were not observed.

### Fungal infections

A total of 52 fungal infection episodes were documented in the study cohort. Candida albicans was the most frequently identified pathogen (14 episodes, 26.9%), followed by Aspergillus fumigatus (5 episodes, 9.6%) and Pneumocystis spp. (5 episodes, 9.6%). Non-albicans Candida species were detected in 4 episodes (7.7%). Cryptococcus neoformans was documented in 2 episodes (3.8%), and other fungi in 3 episodes (5.8%). In one episode (1.9%), the pathogen was not further specified. The median time to fungal infection was 4.6 months (IQR 1.0–15.4 months) after transplantation.

### Factors associated with infectious events

In univariate analyses (Table 5), older donor age was significantly associated with an higher risk of overall infection. Compared with donors aged < 60 years, donor age 60–74 years and ≥ 75 years were both associated with higher infection risk. Higher body mass index and longer inpatient stay were also associated with overall infections.

**Table 5:** Univariable cause-specific Cox regression analyses for infection outcomes and mortality

Outcome	Predictor	Level	HR (95% CI)	p
Overall infection	Age at transplantation	per year	1.00 (0.96–1.04)	0.963
	Male recipient	Male vs Female	0.94 (0.72–1.23)	0.615
	Body mass index	per kg/m^2^	1.04 (1.00–1.07)	0.032
	Deceased donation	Yes vs No	1.26 (0.83–1.92)	0.277
	Male donor	Male vs Female	0.96 (0.74–1.25)	0.726
	Donor age group	60–74 vs < 60	1.85 (1.13–3.03)	0.014
		≥ 75 vs < 60	1.86 (1.12–3.09)	0.016
	CMV mismatch (D +/R −)	Yes vs No	1.31 (0.99–1.73)	0.058
	Cold ischemia time	per minute	1.00 (1.00–1.00)	0.179
	Delayed graft function	Yes vs No	1.07 (0.80–1.43)	0.657
	Inpatient days	per day	1.01 (1.01–1.02)	< 0.001
	Intensified induction therapy	Yes vs No	1.12 (0.82–1.54)	0.465
	Retransplantation	Yes vs No	1.10 (0.74–1.63)	0.629
Bacterial infection	Age at transplantation	per year	1.01 (0.97–1.05)	0.631
	Male recipient	Male vs Female	0.89 (0.67–1.19)	0.392
	Body mass index	per kg/m^2^	1.02 (0.99–1.06)	0.267
	Deceased donation	Yes vs No	1.51 (0.94–2.42)	0.088
	Male donor	Male vs Female	0.97 (0.74–1.28)	0.841
	Donor age group	60–74 vs < 60	1.63 (0.95–2.78)	0.076
		≥ 75 vs < 60	1.79 (1.03–3.11)	0.039
	CMV mismatch (D +/R −)	Yes vs No	1.29 (0.95–1.74)	0.100
	Cold ischemia time	per minute	1.00 (1.00–1.00)	0.840
	Delayed graft function	Yes vs No	1.18 (0.87–1.60)	0.281
	Inpatient days	per day	1.01 (1.01–1.02)	< 0.001
	Intensified induction therapy	Yes vs No	0.90 (0.65–1.25)	0.549
	Retransplantation	Yes vs No	1.06 (0.70–1.60)	0.789
Viral infection	Age at transplantation	per year	1.03 (0.97–1.08)	0.326
	Male recipient	Male vs Female	0.80 (0.57–1.11)	0.189
	Body mass index	per kg/m^2^	0.97 (0.93–1.02)	0.255
	Deceased donation	Yes vs No	1.44 (0.84–2.45)	0.187
	Male donor	Male vs Female	0.95 (0.68–1.33)	0.786
	Donor age group	60–74 vs < 60	1.63 (0.82–3.25)	0.165
		≥ 75 vs < 60	1.50 (0.74–3.04)	0.264
	CMV mismatch (D +/R −)	Yes vs No	1.30 (0.92–1.85)	0.139
	Cold ischemia time	per minute	1.00 (1.00–1.00)	0.952
	Delayed graft function	Yes vs No	0.87 (0.58–1.30)	0.474
	Inpatient days	per day	1.00 (1.00–1.01)	0.309
	Intensified induction therapy	Yes vs No	1.22 (0.81–1.84)	0.338
	Retransplantation	Yes vs No	0.95 (0.59–1.53)	0.843
Fungal infection	Age at transplantation	per year	1.00 (0.92–1.09)	0.976
	Male recipient	Male vs Female	0.66 (0.36–1.22)	0.188
	Body mass index	per kg/m^2^	1.02 (0.95–1.09)	0.542
	Deceased donation	Yes vs No	1.18 (0.42–3.34)	0.747
	Male donor	Male vs Female	0.97 (0.52–1.81)	0.918
	Donor age group	60–74 vs < 60	2.72 (0.65–11.4)	0.173
		≥ 75 vs < 60	1.91 (0.43–8.44)	0.396
	CMV mismatch (D +/R −)	Yes vs No	1.46 (0.76–2.81)	0.255
	Cold ischemia time	per minute	1.00 (1.00–1.00)	0.032
	Delayed graft function	Yes vs No	1.84 (0.97–3.49)	0.063
	Inpatient days	per day	1.02 (1.01–1.03)	< 0.001
	Intensified induction therapy	Yes vs No	1.88 (0.97–3.63)	0.060
	Retransplantation	Yes vs No	1.19 (0.47–3.03)	0.715
Resistant infection	Age at transplantation	per year	1.01 (0.94–1.08)	0.742
	Male recipient	Male vs Female	0.60 (0.35–1.02)	0.061
	Body mass index	per kg/m^2^	1.02 (0.96–1.09)	0.510
	Deceased donation	Yes vs No	1.57 (0.57–4.36)	0.384
	Male donor	Male vs Female	0.68 (0.38–1.22)	0.186
	Donor age group	60–74 vs < 60	2.41 (0.57–10.1)	0.231
		≥ 75 vs < 60	3.95 (0.93–16.8)	0.062
	CMV mismatch (D +/R −)	Yes vs No	1.05 (0.56–1.96)	0.878
	Cold ischemia time	per minute	1.00 (1.00–1.00)	0.437
	Delayed graft function	Yes vs No	1.97 (1.12–3.47)	0.018
	Inpatient days	per day	1.02 (1.01–1.03)	< 0.001
	Intensified induction therapy	Yes vs No	1.14 (0.60–2.18)	0.680
	Retransplantation	Yes vs No	0.62 (0.23–1.73)	0.365
Mortality	Age at transplantation	per year	0.99 (0.90–1.09)	0.862
	Male recipient	Male vs Female	0.75 (0.39–1.44)	0.390
	Body mass index	per kg/m^2^	1.06 (0.98–1.15)	0.174
	Deceased donation	Yes vs No	1.70 (0.52–5.57)	0.381
	Male donor	Male vs Female	0.71 (0.36–1.40)	0.327
	Donor age group	60–74 vs < 60	1.13 (0.33–3.86)	0.844
		≥ 75 vs < 60	1.84 (0.54–6.30)	0.333
	CMV mismatch (D +/R −)	Yes vs No	1.53 (0.77–3.05)	0.226
	Cold ischemia time	per minute	1.00 (1.00–1.00)	0.349
	Delayed graft function	Yes vs No	3.55 (1.88–6.70)	< 0.001
	Inpatient days	per day	1.02 (1.02–1.03)	< 0.001
	Intensified induction therapy	Yes vs No	0.94 (0.39–2.24)	0.886
	Retransplantation	Yes vs No	2.11 (0.92–4.86)	0.077

For bacterial infections, longer inpatient stay was consistently associated with an increased risk. In addition, recipients of kidneys from donors aged ≥ 75 years had a higher risk of bacterial infection compared with those receiving organs from donors aged < 60 years, while deceased donation showed a borderline association.

For viral infections, no significant associations were observed in univariable analyses (Table 5).

For fungal infections, longer inpatient stay was significantly associated with increased risk. Delayed graft function and intensified induction therapy showed borderline associations, whereas cold ischemia time also showed a statistically significant but numerically small effect (Table 5).

For resistant infections, the risk was nearly doubled in patients with delayed graft function and increased further with longer inpatient stay. Borderline associations were observed for male recipient sex and donor age ≥ 75 years (Table 5).

### Factors associated with death with functioning graft (DWFG)

In univariable cause-specific Cox regression analyses for DWFG (Table 6), delayed graft function and longer inpatient stay were associated with a higher risk of subsequent DWFG. Among infectious complications modeled as time-dependent covariates, fungal infection was associated with a markedly increased risk of subsequent DWFG, whereas no significant associations were observed for bacterial or viral infections. When infectious burden was modeled as a cumulative time-dependent exposure, each additional documented infection episode was associated with a 12% increase in the hazard of DWFG.

**Table 6:** Univariable cause-specific Cox regression analyses for death with functioning graft (DWFG)

Predictor	Level	HR (95% CI)	p
Age at transplantation	per year	0.99 (0.90–1.08)	0.862
Male recipient	Male vs Female	0.75 (0.39–1.44)	0.390
Body mass index	per kg/m^2^	1.06 (0.98–1.15)	0.174
Deceased donation	Yes vs No	1.70 (0.52–5.58)	0.381
Male donor	Male vs Female	0.86 (0.44–1.65)	0.640
Donor age group	60–74 vs < 60	1.13 (0.33–3.86)	0.844
	≥ 75 vs < 60	1.84 (0.54–6.28)	0.333
CMV mismatch (D +/R −)	Yes vs No	1.17 (0.57–2.42)	0.670
Cold ischemia time	per minute	1.00 (1.00–1.00)	0.349
Delayed graft function	Yes vs No	2.34 (1.22–4.47)	0.010
Inpatient days	per day	1.03 (1.02–1.04)	< 0.001
Intensified induction therapy	Yes vs No	0.94 (0.39–2.24)	0.886
Retransplantation	Yes vs No	1.75 (0.73–4.22)	0.211
Bacterial infection	Time-dependent	0.73 (0.35–1.55)	0.420
Viral infection	Time-dependent	1.85 (0.82–4.16)	0.140
Fungal infection	Time-dependent	4.03 (1.93–8.44)	< 0.001
Cumulative infectious burden	Per additional infection (time-dependent)	1.12 (1.02–1.23)	0.017

Univariable cause-specific Cox regression analyses for death with functioning graft (DWFG). Infectious complications (bacterial, viral, and fungal infection) were modeled as time-dependent covariates. Cumulative infectious burden was modeled as a time-dependent cumulative count variable, increasing with each documented infection episode during follow-up. HR, hazard ratio; CI, confidence interval; DWFG, death with functioning graft; DGF, delayed graft function; CMV, cytomegalovirus; D +/R −, CMV-seropositive donor/CMV-seronegative recipient

### Multivariate cox regression analyses

In exploratory multivariable cause-specific Cox regression analyses (Table 7), overall infection remained associated with donor age 60–74 years and longer inpatient stay was independently associated with overall infection.

**Table 7:** Multivariable cause-specific Cox regression analyses for infection outcomes and mortality

Outcome	Predictor	Level	Adjusted HR (95% CI)	p
Overall infection	Deceased donation	Yes vs No	1.32 (0.82–2.11)	0.251
	Donor age group	60–74 vs < 60	1.74 (1.06–2.85)	0.029
		≥ 75 vs < 60	1.55 (0.92–2.59)	0.098
	CMV mismatch (D +/R −)	Yes vs No	1.24 (0.92–1.66)	0.153
	Delayed graft function	Yes vs No	1.03 (0.75–1.41)	0.870
	Inpatient days	per day	1.01 (1.00–1.02)	0.007
	Intensified induction therapy	Yes vs No	0.90 (0.65–1.26)	0.556
Bacterial infection	Deceased donation	Yes vs No	1.96 (1.12–3.42)	0.018
	Donor age group	60–74 vs < 60	1.58 (0.92–2.72)	0.095
		≥ 75 vs < 60	1.45 (0.83–2.54)	0.192
	Delayed graft function	Yes vs No	1.14 (0.81–1.59)	0.449
	Inpatient days	per day	1.01 (1.00–1.02)	0.003
	Intensified induction therapy	Yes vs No	0.77 (0.54–1.11)	0.158
Viral infection	Donor age group	60–74 vs < 60	1.51 (0.75–3.04)	0.247
		≥ 75 vs < 60	1.40 (0.68–2.87)	0.363
	CMV mismatch (D +/R −)	Yes vs No	1.26 (0.87–1.82)	0.217
	Intensified induction therapy	Yes vs No	1.12 (0.72–1.73)	0.615
Fungal infection	Delayed graft function	Yes vs No	1.74 (0.91–3.32)	0.095
	Inpatient days	per day	1.01 (1.00–1.02)	0.009
	Intensified induction therapy	Yes vs No	1.65 (0.83–3.29)	0.154
Resistant infection	Deceased donation	Yes vs No	1.62 (0.56–4.64)	0.372
	Delayed graft function	Yes vs No	1.48 (0.83–2.66)	0.187
	Inpatient days	per day	1.02 (1.01–1.03)	< 0.001
Mortality	Delayed graft function	Yes vs No	3.01 (1.56–5.79)	< 0.001
	Inpatient days	per day	1.02 (1.02–1.03)	< 0.001
	Retransplantation	Yes vs No	2.13 (0.91–4.99)	0.080

Adjusted hazard ratios (HR) with 95% confidence intervals (CI) derived from exploratory multivariable cause-specific Cox proportional hazards regression models. Variable inclusion was guided by clinical relevance, univariable associations, and the number of observed events to reduce overfitting. Death before occurrence of the event of interest was treated as a competing event and censored at the time of occurrence. HR, hazard ratio; CI, confidence interval; CMV, cytomegalovirus; D +/R −, CMV-seropositive donor/CMV-seronegative recipient; DGF, delayed graft function

For bacterial infections, deceased transplantation and longer inpatient stay remained independently associated.

For viral infections, no variable retained statistical significance in multivariable analyses (Table 7).

For fungal infections, longer inpatient stay remained associated with increased risk, whereas delayed graft function showed a borderline association.

For resistant infections, only longer inpatient stay remained independently associated.

In multivariable cause-specific Cox regression analyses for DWFG (Table 8), time-dependent fungal infection remained independently associated with a higher risk of subsequent DWFG in the main model, together with delayed graft function and longer inpatient stay.

**Table 8:** Multivariable cause-specific Cox regression analyses for Death With Functioning Graft (DWFG)

Predictor	Level	Adjusted HR (95% CI)	p
Main model			
Fungal infection	Time-dependent	2.67 (1.23–5.77)	0.013
Delayed graft function	Yes vs No	2.54 (1.29–5.00)	0.007
Inpatient days	per day	1.02 (1.02–1.03)	<0.001
CMV mismatch (D+/R−)	Yes vs No	0.74 (0.32–1.69)	0.474
Sensitivity model			
Fungal infection	Time-dependent	2.72 (1.27–5.84)	0.010
Viral infection	Time-dependent	2.50 (1.06–5.91)	0.037
Delayed graft function	Yes vs No	2.58 (1.31–5.06)	0.006
Inpatient days	per day	1.03 (1.02–1.03)	<0.001
CMV mismatch (D+/R−)	Yes vs No	0.69 (0.30–1.59)	0.388

Adjusted for clinically selected covariates. Main model included fungal infection (time-dependent), delayed graft function (DGF), inpatient days, and CMV mismatch (donor-positive/recipient-negative [D+/R−]). Sensitivity model additionally included viral infection as a second time-dependent infectious exposure. HR, hazard ratio; CI, confidence interval; DWFG, death with functioning graft; DGF, delayed graft function; CMV, cytomegalovirus; D+/R−, CMV-seropositive donor/CMV-seronegative recipient.

In the sensitivity model including both fungal and viral infections as time-dependent covariates, associations remained largely unchanged. Fungal infection continued to be associated with an increased risk of DWFG, while viral infection was also associated with subsequent DWFG. Delayed graft function and longer inpatient stay likewise remained independently associated with increased DWFG risk (Table 8). In a separate multivariable analysis incorporating cumulative infectious burden as a time-dependent cumulative count variable together with delayed graft function and inpatient days, the association between cumulative infection burden and DWFG was attenuated and no longer reached statistical significance, while delayed graft function and longer inpatient stay remained independently associated with DWFG.

### Associations with impaired renal function at one year

For the renal outcome analysis, impaired graft function at approximately 1 year after transplantation was common, with 85.4% of patients showing an eGFR < 60 mL/min, 64.0% an eGFR < 45 mL/min, and 32.6% an eGFR < 30 mL/min. In multivariable logistic regression analyses using eGFR < 45 mL/min as the outcome, donor age ≥ 75 years (adjusted OR 4.01, 95% CI 1.44–11.85; *p* = 0.009), older recipient age (adjusted OR 1.10 per year, 95% CI 1.01–1.22; *p* = 0.034), and longer inpatient stay (adjusted OR 1.06 per day, 95% CI 1.02–1.11; *p* = 0.002) were independently associated with impaired renal function. The association for donor age 60–74 years was borderline (adjusted OR 2.61, 95% CI 1.02–7.13; *p* = 0.051). In contrast, the number of infections occurring before renal function assessment was not significantly associated with impaired renal function (adjusted OR 1.06, 95% CI 0.92–1.22; *p* = 0.432).

### Sex-specific outcomes

Cumulative incidences of death, all-cause graft loss, and first infection were similar between female and male recipients. At 5 years, cumulative mortality was 13.5% (95% CI 5.0–21.0) in females and 11.0% (6.0–16.0) in males. Corresponding all-cause graft loss rates were 2.6% (0.0–5.5) in females and 7.7% (3.5–14.1) in males. When graft loss components were analyzed separately, 5-year cumulative incidence of DWFG was 12.3% (95% CI 5.3–19.4) in females and 10.2% (95% CI 5.6–14.8) in males, whereas DCGL was 1.3% (95% CI 0–3.4) and 5.5% (95% CI 2.1–8.9), respectively. The cumulative incidence of first infection reached 84.1% (75.0–90.0) in females and 76.1% (69.0–82.0) in males at 5 years. Resistant infection rates differed between females and males, with 20.0% (23/115) in females and 12.9% (31/240) in males (χ^2^ test, *p* = 0.114).

### Outcome of recipients aged ≥ 70 years

Baseline characteristics were largely comparable between recipients aged 65–69 and ≥ 70 years (*n* = 95, 26.7%), with no clinically relevant differences observed across recipient demographics, donor characteristics, or perioperative variables (Table S3, Figs. 1, 3 and S2).

For bacterial infections, 1-year cumulative incidence was 30.7% (95% CI 24.6–36.3) in patients aged 65–69 years and 36.8% (95% CI 26.1–45.9) in those aged ≥ 70 years. At 3 years, estimates were comparable between groups (70.9% [95% CI 64.3–76.2] vs. 70.4% [95% CI 59.1–78.6]). For viral infections, at 1 year, rates were 7.5% (95% CI 4.0–10.8) in patients aged 65–69 years and 11.4% (95% CI 4.5–17.8) in those aged ≥ 70 years; at 5 years, cumulative incidence reached 32.2% (95% CI 25.1–38.6) and 38.6% (95% CI 26.1–49.0), respectively. CMV demonstrated lower 1-year cumulative incidence in patients aged ≥ 70 years compared with those aged 65–69 years (7.8% vs. 14.5%), while 5-year estimates were comparable (24.9% vs. 22.4%). BKV cumulative incidence was similar between age groups at both 1 year (11.0% vs. 9.7%) and 5 years (11.0% vs. 11.6%). HSV-1 cumulative incidence was higher in patients aged ≥ 70 years at 1 year (3.2% vs. 1.2%) and 5 years (6.0% vs. 3.3%). VZV incidence remained low overall, with minimal differences between age groups (5-year: 3.9% vs. 1.9%).

For fungal infections, 1-year cumulative incidence was 8.1% (95% CI 4.6–11.4) in patients aged 65–69 years and 9.9% (95% CI 3.5–15.8) in those aged ≥ 70 years. At 5 years, estimates were 14.4% (95% CI 9.4–19.1) and 12.6% (95% CI 5.3–19.4), respectively.

Regarding graft-related outcomes, graft loss at 5 years occurred in 6.9% (95% CI 3.0–10.7) of patients aged 65–69 years compared with 4.5% (95% CI 0.1–8.8) in those aged ≥ 70 years. Similarly, death with a functioning graft at 5 years occurred in 11.8% (95% CI 6.9–16.3) and 12.0% (95% CI 3.4–19.9) of patients, respectively. New onset tumor complications were observed in 17.9% (17/95).

## Discussion

In this multicenter cohort study, we provide contemporary real-world data on kidney transplantation in recipients aged ≥ 65 years, a population that is rapidly expanding in routine transplant practice [24]. Our data support that kidney transplantation can be performed successfully in carefully selected older patients, even in the context of predominantly deceased and older donors. These findings are consistent with previous reports indicating that carefully selected older adults can derive substantial benefit from transplantation and that chronological age alone should not be regarded as a contraindication to kidney transplantation [25–28].

At the same time, our data suggest that outcomes in this population are determined by more than merely graft-related factors alone. One of the most notable findings of our study is the pronounced discrepancy between the substantial burden of infectious morbidity and the comparatively lower incidence of hard clinical endpoints. More than half of recipients experienced a first infection within the first year after transplantation, cumulative incidence exceeded 80% during follow-up, and infectious episodes frequently recurred, with a median of three infection episodes per recipient. In contrast, graft loss and mortality remained substantially less frequent. However, death with a functioning graft represented the predominant graft-related endpoint in our cohort, and infections were the leading attributed cause of death with a functioning graft. This indicates that infectious complications do not necessarily follow a catastrophic course, yet are by no means harmless. While many infectious episodes remain clinically manageable, a subgroup of recipients appears to bear a disproportionately high burden of adverse sequelae following an infection. In this sense, the clinically relevant question arising from our data may not be who develops an infection, but rather who lacks the physiological reserve and recovery capacity required to tolerate it.

Notably, not all infectious phenotypes appeared to carry the same clinical signal. Bacterial infections accounted for the majority of infectious episodes and dominated the overall infectious burden [13, 14]. The predominance of urogenital infections likely reflects, at least in part, routine urological instrumentation and perioperative manipulation inherent to kidney transplantation. However, they did not show the same prognostic pattern as fungal infections, which were comparatively rare but strongly associated with subsequent death with a functioning graft. While fungal infections are relatively uncommon after kidney transplantation, it has consistently been associated with substantial morbidity and mortality in previous studies [29–31]. These findings likely reflect both the considerable clinical impact of these infections and the underlying vulnerability of the affected recipients. This pattern is also consistent with previous reports in older kidney transplant recipients showing that competing patient-related risks increasingly outweigh classic transplant-specific failure mechanisms [32, 33].

Our findings further suggest that infection should not be viewed solely as an intermittent complication after transplantation. Rather, the median of three infectious episodes per recipient indicates that infectious morbidity often constitutes a persistent component of the post-transplant experience in older recipients.

Beyond infection-specific findings, postoperative clinical complexity emerged as a recurring signal across several analyses. Delayed graft function and prolonged inpatient stay were repeatedly associated with adverse downstream outcomes, including mortality, graft-related outcomes, resistant infections, and impaired graft function. Similar observations have been reported previously. Tapiawala et al. demonstrated that delayed graft function was independently associated with an increased risk of death with a functioning graft, while de Sandes-Freitas et al. showed that prolonged delayed graft function was associated with both graft loss and mortality [34, 35]. Likewise, longer duration of delayed graft function has been linked to inferior long-term graft and patient outcomes, and prolonged hospitalization after transplantation has been associated with adverse post-transplant outcomes and may capture aspects of postoperative complexity that are not fully reflected by conventional recipient and donor characteristics [36–39]. Together, these findings suggest that the prognostic relevance of early post-transplant recovery extends well beyond the immediate postoperative period. Importantly, length of stay is likely best interpreted not as an isolated modifiable risk factor, but as a composite marker of early post-transplant complexity and recovery.

Collectively, our observations indicate that outcomes in older kidney transplant recipients may increasingly be shaped by recipient-related factors and the clinical course after transplantation rather than by classical transplant-specific failure mechanisms alone. Consistent with this concept, Mayrdorfer et al. reported that among recipients aged > 70 years, medical events accounted for 78% of overall graft losses, whereas only 6.5% were associated with rejection, highlighting the dominant role of non-immunological complications in determining outcomes in older transplant recipients [33]. Similar observations have been reported in other studies of older transplant recipients, in whom death with a functioning graft increasingly exceeds immunological causes as a determinant of long-term outcome [27, 40].

At the same time, baseline demographic strata alone appeared less informative than might be expected. Recipients aged ≥ 70 years showed outcomes broadly comparable to those aged 65–69 years, with no major differences in mortality, graft-related outcomes, or overall infectious burden. Similarly, sex-specific analyses did not reveal major differences in mortality, all-cause graft loss, death with a functioning graft, or first infection, although resistant infections were numerically more frequent among female recipients. These observations suggest that chronological age alone may provide only limited discrimination of risk within an already selected population of older transplant recipients.

This raises an important implication for risk assessment in kidney transplantation in older recipients. The central clinical question may not simply be whether older recipients can achieve acceptable graft outcomes—they often can when carefully selected—but which recipients are likely to successfully navigate the cumulative challenges of the post-transplant course. Our findings suggest that a relevant part of clinically relevant risk may depend on characteristics that are not adequately captured by conventional demographic and clinical variables. This interpretation is consistent with growing evidence that frailty, functional impairment, and reduced physiological reserve provide prognostic information beyond traditional risk factors and are associated with adverse outcomes after kidney transplantation, including mortality, and prolonged hospitalization [36, 38, 39]. Such factors are difficult to assess objectively using routine pre-transplant evaluation and therefore remain largely dependent on subjective clinical impression and physician judgment. Developing objective tools capable of capturing this dimension of risk may therefore represent an important next step for improving risk stratification in older transplant candidates.

The multicenter design and systematic longitudinal assessment of infectious complications represent important strengths of this study, allowing a detailed characterization of infectious burden in an older transplant population that remains underrepresented in current outcome research. However, several limitations warrant consideration. As an observational cohort study, all findings should be interpreted as associations rather than causal effects. Although infectious complications were captured in detail, other relevant post-transplant events, including cardiovascular complications, non-infectious hospitalizations, functional decline, and frailty progression, were not assessed with comparable granularity. Consequently, the relative contribution of infectious complications to overall post-transplant morbidity remains uncertain. In addition, some subgroup analyses were limited by small event numbers, residual confounding cannot be excluded, and some data remained incomplete despite additional efforts, reflecting the inherent limitations of multicenter real-world data collection.

## Conclusion

Kidney transplantation in carefully selected recipients aged ≥ 65 years, including septuagenarians, was associated with acceptable graft-related and patient-related outcomes. Infectious complications represented a substantial component of post-transplant morbidity and, although often clinically manageable, could entail severe consequences in a vulnerable subgroup of recipients, particularly in the context of death with a functioning graft. Future studies should focus on developing objective approaches to identify physiological vulnerability, resilience, and recovery capacity in older transplant candidates in order to readily complement existing clinical assessment and improve individualized risk stratification.

## Supplementary Information

Supplementary Material 1.

## Data Availability

No datasets were generated or analysed during the current study.

## Abbreviations

CI: Confidence interval
CKD-EPI: Chronic Kidney Disease
DWFG: Death with functioning graft
DCGL: Death-censored graft loss
CMV: Cytomeglovirus
EBV: Epstein-Barr Virus
eGFR: Estimated glomerular filtration rate
GFR: Glomerular filtration rate
HR: Hazard ratio
IQR: Interquartile range
KDIGO: Kidney Disease Improving Global Outcomes
MDRD: Modification of Diet in Renal Disease
RSV: Respiratory syncytial virus
SARS-CoV2: Severe acute respiratory syndrome coronavirus 2
