# Supplementary material for: Renal transplantation in older recipients – results of the DZIF transplant cohort
Source: BMC Geriatr. 2026 Jul 6;26:916. doi: 10.1186/s12877-026-07901-0 (PMC13343768; doi:10.1186/s12877-026-07901-0)
Supplement: Supplementary file 1 — Supplementary Material 1. [file 12877_2026_7901_MOESM1_ESM.docx]

**Supplementary Material**

**Supplementary Table S1. Definitions of infectious complications**

| **Category** | **Infection type** | **Operational definition** |
| --- | --- | --- |
| General definition | Infectious episode | Clinically suspected or microbiologically confirmed infection requiring hospitalization and systemic anti-infective therapy (antibiotic, antiviral, or antifungal). Diagnosis documented by the treating physician. Separate episodes were defined if occurring ≥14 days after completion of prior therapy and after documented clinical resolution. |
| Bacterial infections | Urinary tract infection (UTI) | Clinical symptoms (e.g., dysuria, urgency, flank pain, fever) and urine culture ≥10⁵ CFU/mL of a pathogenic organism, or physician diagnosis requiring systemic antibiotic therapy. Asymptomatic bacteriuria was not classified as infection. |
|  | Bloodstream infection (BSI) | ≥1 positive blood culture for a pathogenic organism, or ≥2 positive cultures for common contaminants plus compatible clinical signs. |
|  | Respiratory tract infection | New pulmonary infiltrate on imaging plus compatible clinical symptoms (fever, cough, dyspnea); microbiological confirmation when available. |
|  | Gastrointestinal infection | Clinical symptoms (e.g., diarrhea, abdominal pain) requiring hospitalization, with microbiological and/or endoscopic confirmation when available. |
|  | Surgical site infection | Superficial, deep, or organ/space infection consistent with CDC criteria, requiring systemic therapy and/or intervention. |
|  | Catheter-associated infection | Infection temporally associated with indwelling urinary or vascular catheter use, supported by clinical findings and/or microbiology, requiring systemic therapy. |
| Viral infections | CMV infection/disease | CMV DNAemia detected by quantitative PCR requiring intravenous antiviral therapy (e.g., ganciclovir) during hospitalization, with or without clinical symptoms. Tissue-invasive CMV confirmed histologically was also included. Asymptomatic DNAemia not requiring intravenous treatment was excluded. |
|  | BK virus (BKV) | BKV DNAemia detected by PCR above institutional threshold requiring clinical management during hospitalization. |
|  | HSV-1 / HSV-2 | Compatible mucocutaneous or systemic disease requiring hospitalization; PCR or culture confirmation when available. |
|  | Varicella zoster virus (VZV) | Clinical diagnosis of herpes zoster or varicella requiring hospitalization; PCR confirmation when available. |
|  | Epstein–Barr virus (EBV) | EBV DNAemia requiring hospitalization or EBV-positive post-transplant lymphoproliferative disorder (PTLD) confirmed histologically. |
|  | Respiratory viral infection | Positive PCR from respiratory sample with compatible symptoms requiring hospitalization. |
| Fungal infections | Candida infection | Proven infection (culture from sterile site) or symptomatic infection requiring systemic antifungal therapy during hospitalization. |
|  | Aspergillus infection | Clinically suspected or microbiologically detected Aspergillus spp. infection requiring systemic antifungal therapy during hospitalization, based on physician documentation. |
|  | Pneumocystis jirovecii pneumonia (PJP) | Compatible clinical presentation with PCR and/or microscopy from respiratory sample, requiring treatment and hospitalization. |
|  | Cryptococcus neoformans | Positive culture or antigen detection from sterile site, requiring systemic antifungal therapy. |

**Table S2. Frequency and burden of infection episodes according to pathogen category**

| **Infection type** | **Patients with ≥1 episode, n (%)** | **Episodes per 100 patient-years** | **Median episodes per patient (IQR)** |
| --- | --- | --- | --- |
| **Any infection** | 257 (72.4) | 63.9 | 3 (2–5) |
| **Bacterial infection** | 215 (60.6) | 42.0 | 2 (1–3) |
| **Viral infection** | 157 (44.2) | 18.7 | 1 (0–2) |
| **Fungal infection** | 40 (11.3) | 3.2 | 0 (0–0) |
| Infection rates were calculated as the number of documented infection episodes per 100 patient-years of follow-up (total follow-up: 1,468 patient-years). Median numbers of infection episodes per patient are presented with interquartile range (IQR). Patients could contribute to more than one pathogen category. | | | |

**Table S3.** Baseline characteristics by recipient age group.

| **Characteristic** | **65–69 years N = 260** | **≥70 years N = 95** |
| --- | --- | --- |
| **Recipient Characteristics** | | |
| Age at transplantation, years | 67.0 (65.0–68.0) | 72.0 (71.0–75.0) |
| Male gender | 173 (66.5%) | 67 (70.5%) |
| Body mass index, kg/m² | 26.0 (23.5–29.0) | 25.8 (24.0–27.9) |
| Glomerulonephritis (combined) | 76 (29.5%) | 21 (23.6%) |
| ADPKD | 38 (15%) | 13 (15%) |
| Diabetes mellitus type 1 | 7 (2.8%) | 1 (1.1%) |
| Diabetes mellitus type 2 | 14 (5.6%) | 9 (10%) |
| FSGS | 14 (5.6%) | 4 (4.5%) |
| Vasculitis / collagen vascular disease | 9 (3.5%) | 4 (4.4%) |
| Times transplanted: 0 | 218 (88%) | 84 (93%) |
| Times transplanted: 1 | 27 (11%) | 6 (6.7%) |
| Times transplanted: 2 | 4 (1.6%) | 0 (0%) |
| Recipient Serostatus |  |  |
| CMV IgG positive | 145 (58%) | 53 (56%) |
| EBV IgG positive | 216 (94%) | 74 (87%) |
| **Transplant characteristics** | | |
| Multi-organ transplantation (pancreas–kidney) | 5 (1.9%) | 3 (3.2%) |
| ABO incompatible | 4 (1.6%) | 6 (6.7%) |
| Deceased donation | 238 (91.5%) | 78 (82.1%) |
| Cold ischemia time, min | 583.0 (412.0–765.0) | 482.0 (339.0–696.0) |
| **Donor characteristics** | | |
| Donor male gender | 100 (43%) | 34 (38%) |
| 15 to <20 | 1 (0.4%) | 0 (0%) |
| 20 to <25 | 2 (0.9%) | 1 (1.1%) |
| 30 to <35 | 2 (0.9%) | 0 (0%) |
| 35 to <40 | 2 (0.9%) | 0 (0%) |
| 40 to <45 | 1 (0.4%) | 0 (0%) |
| 45 to <50 | 4 (1.7%) | 1 (1.1%) |
| 50 to <55 | 6 (2.6%) | 2 (2.2%) |
| 55 to <60 | 7 (3.0%) | 2 (2.2%) |
| 60 to <65 | 16 (6.8%) | 9 (10%) |
| 65 to <70 | 65 (28%) | 25 (28%) |
| 70 to <75 | 50 (21%) | 14 (16%) |
| 75 to <80 | 41 (18%) | 20 (22%) |
| 80 or older | 37 (16%) | 16 (18%) |
| CMV IgG positive | 161 (63%) | 50 (53%) |
| EBV IgG positive | 191 (74%) | 75 (79%) |
| D+/R+ | 94 (37.3%) | 31 (33.3%) |
| D+/R− | 62 (24.6%) | 19 (20.4%) |
| D−/R+ | 52 (20.6%) | 22 (23.7%) |
| D−/R− | 44 (17.5%) | 21 (22.6%) |
| **Induction therapy** | | |
| intensified induction therapy* | 52 (20.8%) | 17 (18.3%) |
| Thymoglobuline | 29 (11.2%) | 10 (10.5%) |
| Plasmapheresis | 13 (5.0%) | 6 (6.3%) |
| **Early post-transplant outcomes** | | |
| Delayed graft function (DGF) | 64 (24.6%) | 21 (22.1%) |
| Inpatient days | 19.0 (14.0–27.0) | 19.0 (15.0–29.0) |
| Cold ischemia time, min | 583.0 (412.0–765.0) | 482.0 (339.0–696.0) |
| IMC days | 5.0 (4.0–6.0) | 5.0 (4.0–6.0) |
| Data are presented as median (interquartile range) for continuous variables and n (%) for categorical variables. Percentages refer to column totals. Analyses were performed using a complete-case approach; no imputation of missing data was performed. *Intensified induction therapy included antithymocyte globulin (Thymoglobuline), rituximab, and/or plasmapheresis.  Abbreviations: ADPKD, autosomal dominant polycystic kidney disease; FSGS, focal segmental glomerulosclerosis; CMV, cytomegalovirus; EBV, Epstein–Barr virus; DGF, delayed graft function; IMC, intermediate care unit; IMC, intermediate care unit; D, donor; R, recipient. | | |


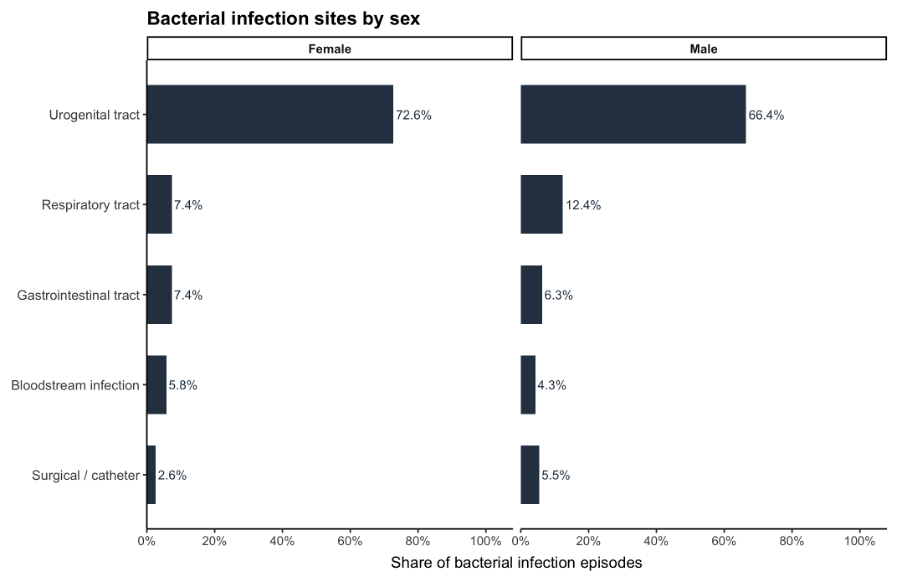


**Figure S1.** Bacterial infection sites stratified by sex.
Relative distribution of bacterial infection episodes by anatomical site, stratified by sex. Percentages are calculated within each sex.


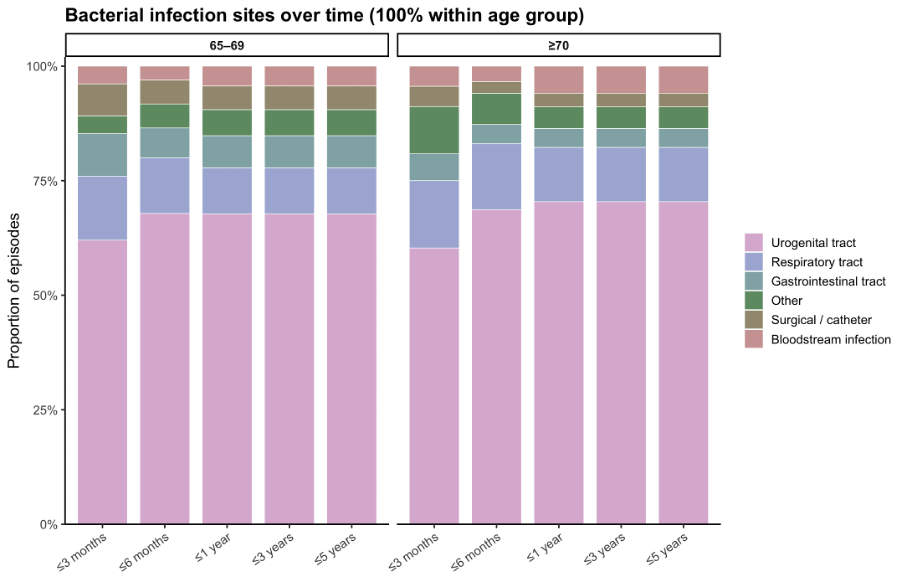


**Figure S2.** Bacterial infection sites stratified by age groups 65-69 years and ≥70 years.
